# Supplementary material for: A-REST (Activity to Reduce Excessive Sitting Time): A Feasibility Trial to Reduce Prolonged Sitting in Police Staff
Source: Int J Environ Res Public Health. 2022 Jul 27;19(15):9186. doi: 10.3390/ijerph19159186 (PMC9368451; doi:10.3390/ijerph19159186)
Supplement: Supplementary file 1 [file ijerph-19-09186-s001.zip › ijerph-1789158-supplementary.pdf]

Supplementary file 1. CONSORT checklist for the A-REST study.

CONSORT 2010 checklist of information to include when reporting a pilot or feasibility trial*

Section/ Topic	Item No	Checklist item	Reported on page No
**Title and abstract**			
	1a	Identification as a pilot or feasibility randomised trial in the title	TITLE
	1b	Structured summary of pilot trial design, methods, results, and conclusions (for specific guidance see CONSORT abstract extension for pilot trials)	ABSTRACT
**Introduction**			
Background and objectives	2a	Scientific background and explanation of rationale for future definitive trial, and reasons for randomised pilot trial	INTRODUCTION
	2b	Specific objectives or research questions for pilot trial	INTRODUCTION
**Methods**			
Trial design	3a	Description of pilot trial design (such as parallel, factorial) including allocation ratio	DESIGN & FIGURE 1
	3b	Important changes to methods after pilot trial commencement (such as eligibility criteria), with reasons	RESULTS
Participants	4a	Eligibility criteria for participants	ELIGIBILITY CRITERIA
	4b	Settings and locations where the data were collected	STUDY SETTING
	4c	How participants were identified and consented	RECRUITMENT, RESULTS
Interventions	5	The interventions for each group with sufficient details to allow replication, including how and when they were actually administered	INTERVENTION, FIGURE 2 (INFOGRAPHIC), FIGURE 3 (CONSORT FLOWCHART), & TIDIER (ADDITIONAL FILE 1: SUPPLEMENTS 2,3,4,5,6)
Outcomes	6a	Completely defined prespecified assessments or measurements to address each pilot trial objective specified in 2b, including how and when they were assessed	OUTCOMES, TABLE 1, ADDITIONAL FILE 1: SUPPLEMENT 8
	6b	Any changes to pilot trial assessments or measurements after the pilot trial commenced, with reasons	N/A
	6c	If applicable, prespecified criteria used to judge whether, or how, to proceed with future definitive trial	TABLE 1
Sample size	7a	Rationale for numbers in the pilot trial	RECRUITMENT
	7b	When applicable, explanation of any interim analyses and stopping guidelines	N/A
Randomisation:			
Sequence generation	8a	Method used to generate the random allocation sequence	N/A
	8b	Type of randomisation(s); details of any	N/A

		restriction (such as blocking and block size)	
Allocation concealment mechanism	9	Mechanism used to implement the random allocation sequence (such as sequentially numbered containers), describing any steps taken to conceal the sequence until interventions were assigned	N/A
Implementation	10	Who generated the random allocation sequence, who enrolled participants, and who assigned participants to interventions	N/A
Blinding	11a	If done, who was blinded after assignment to interventions (for example, participants, care providers, those assessing outcomes) and how	N/A
	11b	If relevant, description of the similarity of interventions	N/A
Statistical methods	12	Methods used to address each pilot trial objective whether qualitative or quantitative	ANALYSES
**Results**			
Participant flow (a diagram is strongly recommended)	13a	For each group, the numbers of participants who were approached and/or assessed for eligibility, randomly assigned, received intended treatment, and were assessed for each objective	PARTICIPATION RATES AND SAMPLE DESCRIPTION; FIGURE 3 (CONSORT FLOWCHART)
	13b	For each group, losses and exclusions after randomisation, together with reasons	FIGURE 3 (CONSORT FLOWCHART)
Recruitment	14a	Dates defining the periods of recruitment and follow-up	FIGURE 1
	14b	Why the pilot trial ended or was stopped	N/A
Baseline data	15	A table showing baseline demographic and clinical characteristics for each group	TABLE 2
Numbers analysed	16	For each objective, number of participants (denominator) included in each analysis. If relevant, these numbers should be by randomised group	PRIMARY OUTCOMES; TABLE 1
Outcomes and estimation	17	For each objective, results including expressions of uncertainty (such as 95% confidence interval) for any estimates. If relevant, these results should be by randomised group	N/A
Ancillary analyses	18	Results of any other analyses performed that could be used to inform the future definitive trial	SECONDARY OUTCOMES; TABLES 3,4,5; ADDITIONAL FILE 1: SUPPLEMENT 8
Harms	19	All important harms or unintended effects in each group (for specific guidance see CONSORT for harms)	PRIMARY OUTCOMES
	19a	If relevant, other important unintended consequences	DISCUSSION-UNINTENDED EFFECTS
**Discussion**			
Limitations	20	Pilot trial limitations, addressing sources of potential bias and remaining uncertainty about feasibility	STRENGTHS & LIMITATIONS
Generalisability	21	Generalisability (applicability) of pilot trial methods and findings to future definitive trial	DISCUSSION; STRENGTHS &

		and other studies	LIMITATIONS
Interpretation	22	Interpretation consistent with pilot trial objectives and findings, balancing potential benefits and harms, and considering other relevant evidence	DISCUSSION
	22a	Implications for progression from pilot to future definitive trial, including any proposed amendments	DISCUSSION; CONCLUSION
**Other information**			
Registration	23	Registration number for pilot trial and name of trial registry	STUDY DESIGN
Protocol	24	Where the pilot trial protocol can be accessed, if available	BRIERLEY, M.L. CHANGING SEDENTARY BEHAVIOUR IN THE WORKPLACE [DOCTORAL THESIS]. *UNIVERSITY OF BEDFORDSHIRE* **2020**.
Funding	25	Sources of funding and other support (such as supply of drugs), role of funders	FUNDING
	26	Ethical approval or approval by research review committee, confirmed with reference number	STUDY DESIGN; INSTITUTIONAL REVIEW BOARD STATEMENT

Citation: Eldridge SM, Chan CL, Campbell MJ, Bond CM, Hopewell S, Thabane L, et al. CONSORT 2010 statement: extension to randomised pilot and feasibility trials. BMJ. 2016;355.
*We strongly recommend reading this statement in conjunction with the CONSORT 2010, extension to randomised pilot and feasibility trials, Explanation and Elaboration for important clarifications on all the items. If relevant, we also recommend reading CONSORT extensions for cluster randomised trials, non-inferiority and equivalence trials, non-pharmacological treatments, herbal interventions, and pragmatic trials. Additional extensions are forthcoming: for those and for up to date references relevant to this checklist, see www.consort-statement.org.

TIDieR

A-REST (Activity to Reduce Excessive Sitting Time in police staff)

Why:	The primary aim of this study is to assess the feasibility and acceptability of an intervention to reduce and break up prolonged sitting time in full time, adult (18+) police staff. The secondary aims of this study are to assess potential effects on patterns of sedentary behaviour (number of breaks, number of prolonged sitting bouts, average duration of prolonged sitting bouts, and total prolonged sitting duration), additional measures of sedentary behaviour (total sitting time, standing, and stepping), cardiometabolic risk markers, physical health (self-report), psychological wellbeing and mood, work stress (self-report), and work performance (job satisfaction and productivity). The development of the current intervention follows the steps outlined by the Behaviour Change Wheel (Michie et al. 2014). Twenty behaviour change techniques (see Supplementary file 4) were selected for the intervention based on prior research (Brierley et al., 2019, 2021, 2022).
What (material):	BEHAVIOUR CHANGE BOOKLET<br>A behaviour change booklet was developed and adapted from the Active Herts project (ActiveHerts.org.uk) and provides information on health consequences, goal setting, implementation intentions and additional behaviour change techniques related to breaking up and reducing sitting time (see Supplementary file 4). Intervention group participants will receive a physical copy of the booklet (size A5, in colour).<br><br>QR CODES<br>QR codes will be mounted on laminated, size A5 card, printed in colour (see Supplementary file 5). These will be adhered to the wall at chest height at various locations in the building near intervention clusters. QR codes will be located such that they facilitate a three-minute break (including the time to scan and log the break). Once locations have been decided jointly by participants and the researchers (during the education session), an electronic map (pdf) will be emailed to participants by health champions to designate where these codes are located.<br><br>RISE & RECHARGE® APP<br>The Rise & Recharge® smartphone app (Baker Institute, Melbourne, Australia) individually and automatically monitors, records and rewards breaks from sitting with a visual diagram and earned stars presented in real time. The app also provides information on health consequences. The prompting function of the app is not functional during the study.<br><br>EMAILS<br>Weekly email correspondence sent from the health champions will include the weekly leaderboard standings for teams and a personalised support message (see Supplementary file 6). The emails will also let the participants know what the timeline is of the intervention.<br><br>ELECTRONIC PROMPTS<br>A single-sided handout with a list of free computer and smartphone apps (iPhone and Android-compatible choices) will be distributed to participants during the education session.
What (procedures):	The intervention group will aim to regularly break up their prolonged sitting time with three-minute incidental movement breaks every half hour at work. The intervention will support this behavioural change with a short educational lecture, a brainstorming workshop, a behaviour change booklet, computer break prompts, QR codes to log breaks, use of the Rise & Recharge® app for self-monitoring and individual feedback on behaviour, team competition, health champions, and weekly email communication.<br><br>EDUCATION SESSION<br>An interactive education session will be delivered in person by the research lead (MLB) to intervention participants after baseline measurements have been taken. The researcher will deliver a Powerpoint presentation (Microsoft Corporation, Redmond, WA, USA) that exactly matches the behaviour change booklet. BCTs to be delivered include providing information on health consequences, instruction on how to perform the behaviour, demonstration of the

	behaviour, setting common (team) goals, and completion of the behaviour change booklet. It should be noted that the intervention may vary slightly depending on goals set by participants during the session. These changes are not anticipated to significantly alter the intervention but might include things like the exact positioning of QR codes (locations to be decided on jointly by participants and the researchers), the point value of codes, and whether to incorporate breaks during meeting times. Health champions will stay an additional 15 minutes to receive training in how to personalise and send the weekly emails (see 'HEALTH CHAMPIONS' below for details). It may be necessary to accommodate shift patterns with a second lecture.<br><br>BEHAVIOUR CHANGE BOOKLET<br>To be completed individually during the education session.<br><br>COMPUTER PROMPTS<br>Participants will set electronic prompts to remind them to break up their sitting time at work. Prompts will ideally be set to every 30 minutes but may vary according to individual and team goals.<br><br>QR CODES<br>Each QR code will be linked to a unique Google form (Google LLC, Mountain View, CA, USA) where participants will enter their study ID number to log their break from sitting. Each logged break earns a point for the participant's team.<br><br>HEALTH CHAMPIONS<br>A person will volunteer from each team of three to serve as a point of contact for questions from team members, to personalise and send weekly emails, and act as a role model. Health champions will receive additional training after the education session (15 minutes) with regards to setting up computer prompts, setting up their team email contact list, and personalising the weekly emails. NOTE: The intervention was modified to not have health champions, but instead, utilise the research liaison for email personalisation during the intervention (fidelity).<br><br>TEAM COMPETITION<br>Team competition will take place to reward participation with a trophy. Each QR code will be worth points and the winning team will be determined by total points earned every week. All participants will be informed of their team's standing via the weekly emails. The trophy will go to whichever team is in the lead each week. The leading team in week 8 will get to keep the trophy.<br><br>EMAILS<br>Weekly email correspondence sent from the health champions will include the weekly leaderboard standings for teams and a personalised support message. The emails will also let the participants know what the timeline is of the intervention. Health champions will be reminded by the research team (via email or text message) to email to their team every week. Researchers will be copied in on the emails to monitor fidelity. NOTE: The research liaison (who was not a participant in the study) personalised two emails and sent out only one due to time constraints.The remainder were personalised and sent by the research team.
Who provided:	The intervention elements of education session and email reminders to health champions, will be delivered by the research lead (MLB), a PhD candidate with experience in sedentary behaviour change. Pre/post assessments will be undertaken by the laboratory-trained research team (students and staff in the Institute for Sport and Physical Activity Research (ISPAR) at the University of Bedfordshire).
How (mode of delivery; individual or group):	BREAKS FROM PROLONGED SITTING - Performed individually by participants.<br><br>EDUCATION SESSION - Face-to-face delivery to group.<br><br>QR CODES - Physical delivery; individual.

	BEHAVIOUR CHANGE BOOKLET - Physical copy. Individual delivery (participants receive and complete their own booklet).<br><br>ELECTRONIC PROMPTS - Digital delivery; individual.<br><br>RISE & RECHARGE® APP - Digital delivery; individual.<br><br>HEALTH CHAMPIONS - N/A.<br><br>TEAM COMPETITION - N/A<br><br>EMAILS - Digital delivery; group email.
Where:	The intervention will take place at the offices of Bedfordshire Police Force. To prevent cross-contamination, intervention and control clusters will be located in different teams and physically separated by walls, floors or buildings. Office types of participants (e.g., open plan) will be recorded in the demographic survey and presented in the results. NOTE: There was no control group in the actual intervention.
When and how much:	The intervention will be 10-weeks long. Before the first week of the intervention begins, there will be an education session (~1 hour) followed by health champion training (15 minutes). Participants will be asked to take a 3-minute break every half hour at work. Each week of the intervention participants will receive an email with leaderboard standings and a personalised support message (10 emails in total). NOTE: The actual intervention was adjusted to 8 weeks (and 8 emails) and there was no health champion training as planned.
Tailoring:	This intervention is designed to be flexible and support individual pathways to behaviour change. In other words, all participants will be provided the same materials, the same support, and same core set of BCTs, but how they choose to engage with these is up to them. For example, some participants may choose to ignore the computer prompts because they prefer to remember to take a break on their own. These kinds of adaptations are anticipated and will be assessed in exit interviews.
Modification:	Yes, modifications are noted above in the relevant sections.
How well (planned):	Fidelity will be assessed in the Implementation indicator of the RE-AIM framework. Bellg et al. (2004) identified five theoretical elements to intervention fidelity: study design, training of the provider(s), delivery by the provider, receipt of the intervention, and enactment of the behaviour. This framework not only complements feasibility reporting but will also be useful for interpretation of the potential effects of the study.
How well (actual):	The majority of the intervention was delivered as planned. A discussion of this can be found in the Results and Discussion section of the A-REST feasibility paper.

Adapted from Hoffmann T, Glasziou P, Boutron I, Milne R, Perera R, Moher D, Altman D, Barbour V, Macdonald H, Johnston M, Lamb S, Dixon-Woods M, McCulloch P, Wyatt J, Chan A, Michie S. Better reporting of interventions: template for intervention description and replication (TIDieR) checklist and guide. BMJ. 2014;348:g1687.

Supplementary file 3. Table showing behaviour change techniques (BCTs) delivered by A-REST intervention components.

Number	BCT	Education session	Booklet	QR code	Rise & Recharge app	Electronic prompt	Team com-petition	Emails	Teams
1.1	Goal setting (behaviour)*✢^	Y	Y		Y				
1.2	Problem solving*✢	Y	Y						
1.4	Action planning^	Y	Y						
2.1	Monitoring of behaviour by others without feedback			Y					
2.2	Feedback on behaviour✢				Y				
2.3	Self-monitoring of behaviour^				Y				
3.1	Social support (unspecified)✢^	Y							Y
4.1	Instruction on how to perform the behaviour✢^	Y	Y						
5.1	Information about health consequences^	Y	Y		Y				
5.6	Information about emotional consequences		Y						
6.1	Demonstration of the behaviour*	Y							
6.2	Social comparison*✢^						Y		
7.1	Prompts/cues✢^				Y	Y		Y	
8.2	Behaviour substitution*✢^				Y	Y			
8.4	Habit reversal*✢^				Y	Y			
9.1	Credible source✢		Y		Y				
9.2	Pros and cons		Y						
10.3	Non-specific reward✢				Y				
10.4	Social reward✢							Y	
12.2	Restructuring the social environment✢^								Y

* denotes BCTs identified in: Brierley, M.L.; Chater, A.M.; Smith, L.R.; Bailey, D.P. The effectiveness of sedentary behaviour reduction workplace interventions on cardiometabolic risk markers: a systematic review. *Sport Med* **2019**, *49*, doi:10.1007/s40279-019-01168-9.

✢ denotes BCTs evaluated in: Brierley, M.L.; Smith, L.R.; Bailey, D.P.; Ojo, S.O.; Hewson, D.J.; Every, S.A.; Staines, T.A.; Chater, A.M. Evaluating a multi-component intervention to reduce and break up office workers' sitting with sit-stand desks using the APEASE criteria. *BMC Public Health* **2022**, *22*, 458, doi:10.1186/s12889-022-12794-w.

^ denotes BCTs identified in: Brierley, M.L.; Smith, L.R.; Bailey, D.P.; Every, S.A.; Staines;, T.A.; Chater, A.M. Perceived influences on reducing prolonged sitting in police staff: a qualitative investigation using the Theoretical Domains Framework and COM-B model. *BMC Public Health* **2021**, *21*, 1–12.

Supplementary file 4. Behaviour change booklet

BROUGHT TO YOU BY

University of Bedfordshire

a-rest

activity to reduce excessive sitting time

YOUR PERSONAL PLAN TO SIT LESS AT WORK

ABOUT A-REST

A-REST (Activity to Reduce Excessive Sitting Time) is an intervention specifically designed for police staff to help make small, lasting changes to the way you work. The idea is to incorporate short, frequent breaks throughout the workday.

Habits are a big part of this and changing them will help to improve your health and wellbeing.

Keeping in touch

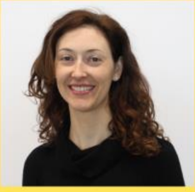

Marsha Brierley
[phone number]
[email address]

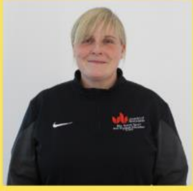

Dr Lindsey Smith
[email address]

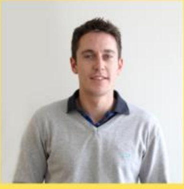

Dr Daniel Bailey
[email address]

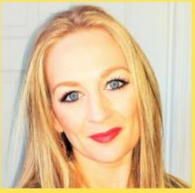

Dr Angel Chater
[email address]

2

IS REDUCING YOUR SITTING TIME GOOD FOR YOU?

Consider the possible positives and negatives of breaking up and reducing your sitting time throughout the workday by filling in the boxes.

Pros

Cons

On a scale of 1 – 10, how confident do you feel about reducing your prolonged sitting?

1 Not at all — 10 Very

YOUR THOUGHTS

About sitting time at work

HURDLES TO JUMP OVER

OVERCOMING YOUR BARRIERS

Think about your current situation. What things in your life might be hurdles that stop you from breaking up and reducing your sitting time at work?

WHAT'S CURRENTLY STOPPING YOU?	HOW CAN YOU OVERCOME THIS?
•	•
•	•
•	•

5

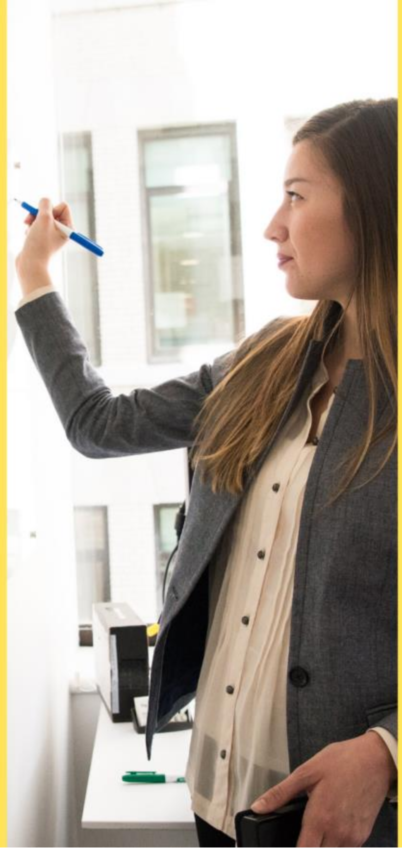

NEW HABITS

Make it a routine

Create a timetable or mindmap to identify opportunities in your day/week to reduce how long you sit at any one time. Start small and slowly build up a solid routine. Think about how you could do something different.

Remember sitting less than 30-mins at a time will start to make a big difference!

6

“I'm doing it
to look after my health”

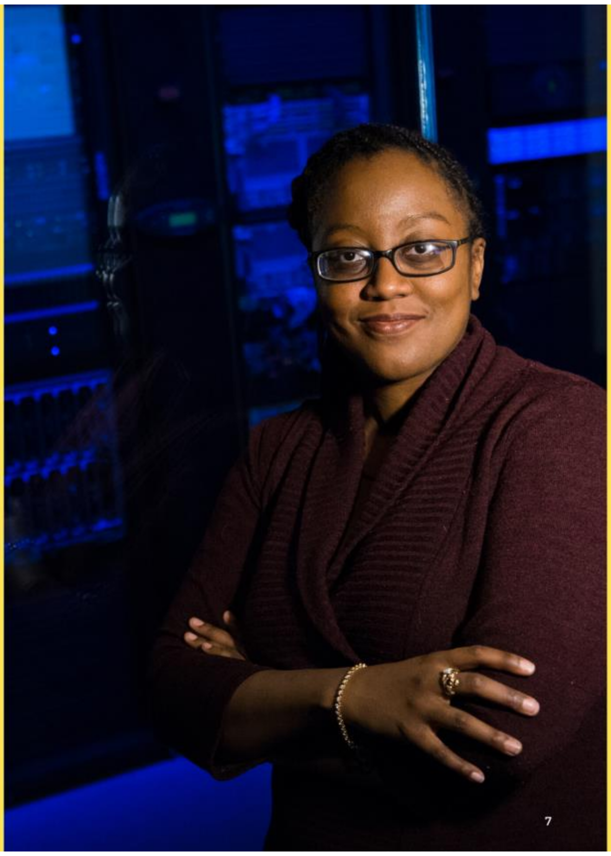

7

Get focused

SET YOUR GOALS AND WORK TOWARDS THEM

Breaking up and reducing your sitting at work can seem like a really tough challenge. The key is to think about what you want to achieve and how you are going to do it. This task will help you to set your goals over the short and medium term.

What do you want to achieve? | How will you achieve it?

Short term
(2 weeks)

How confident are you in achieving this goal? Not at all 1-2-3-4-5-6-7-8-9-10 Very

Medium term
(2 months)

How confident are you in achieving this goal? Not at all 1-2-3-4-5-6-7-8-9-10 Very

8

How to change

YOUR NEXT STEPS FOR SUCCESS

This action plan is your quick guide to success. Make a note of the things you are going to do to break up and reduce your sitting time at work.

	What am I going to do?	Where am I going to do it?	When am I going to do it?	Who am I going to do it with?
○				
○				
○				

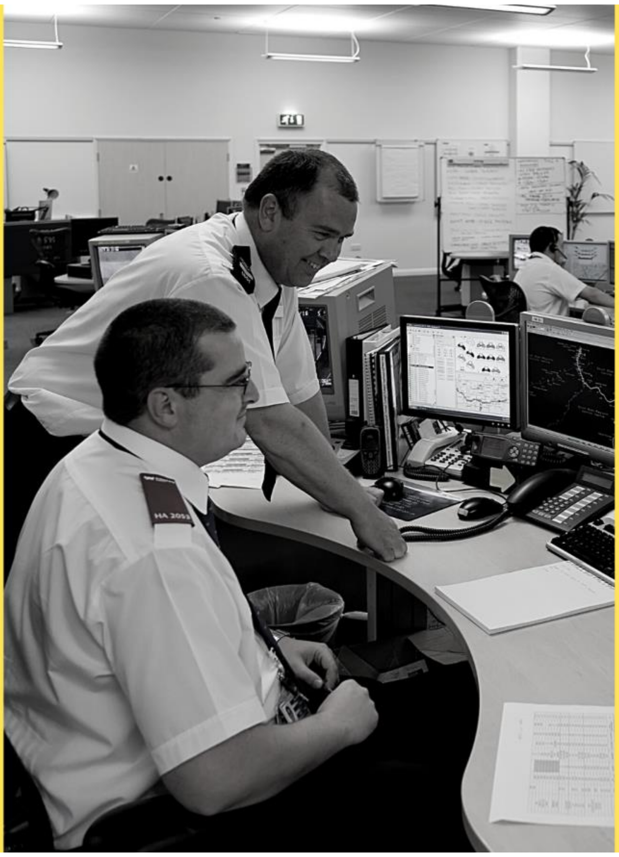

“I'm doing it to improve my blood pressure”

PLANNING IS KEY

BUMPS IN THE ROAD

Breaking up and reducing sitting time at work is not a smooth ride for anyone. This task will help you plan how to avoid or cope with your bumps in the road and increase your chance of success. Think of difficult situations and how you might avoid/cope with them.

IF...	THEN I WILL...
•	•
•	•
•	•

11

Benefits of reducing your sitting time

DID YOU KNOW THAT BREAKING UP YOUR SITTING TIME AT WORK CAN...

- HELP YOU MANAGE HIGH BLOOD PRESSURE
- MAKE YOU FEEL GOOD AND IMPROVE YOUR MENTAL HEALTH
- IMPROVE THE HEALTH OF YOUR HEART AND LOWER YOUR RISK OF A HEART ATTACK OR STROKE
- PREVENT DIABETES AND HELP YOU MANAGE IT BETTER
- GIVE YOU MORE ENERGY
- HELPS YOU MEET NEW PEOPLE
- HELP YOU TO MANAGE YOUR WEIGHT

12

SITTING GUIDELINES

WHAT YOU SHOULD BE AIMING FOR

BREAK UP YOUR SITTING

- Take frequent, short (3-min) breaks throughout the day
- Very light intensity activities count as breaks
- Aim for 16 breaks in an 8-h workday
- Sit for less than 30-mins at a time

WHAT COUNTS AS A 'BREAK'?

- A walk to the kitchen or toilets
- Standing to make a cup of tea or coffee
- Pacing while on the phone
- Walking over to talk to a colleague
- Stretching
- Taking the stairs

13

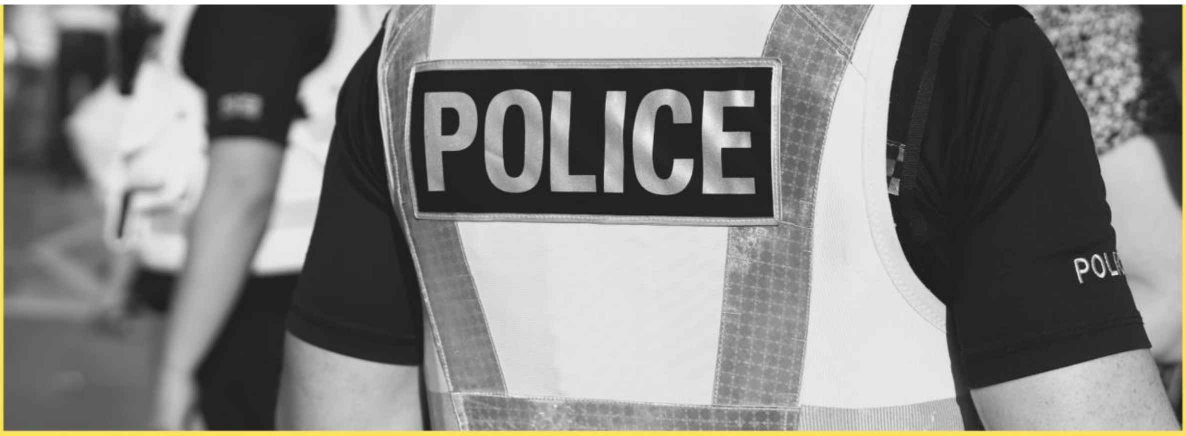

“I'm doing it to inspire others”

14

This is where your path begins

01

WORKSHOP

HERE YOU'LL LEARN ABOUT THE STUDY AND ABOUT THE HEALTH BENEFITS TO BREAKING UP AND REDUCING EXCESSIVE SITTING TIME AT WORK

WORK THROUGH THIS BOOKLET TO SET INDIVIDUAL GOALS

COLLABORATE WITH YOUR COLLEAGUES TO SET TEAM GOALS

ASK QUESTIONS AND GET MOTIVATED!

02

10-WEEK INTERVENTION

THE AIM OF THE STUDY IS TO HELP YOU REDUCE YOUR SITTING TIME WITH SHORT, FREQUENT BREAKS THROUGHOUT THE WORKDAY (3MINS EVERY HALF HOUR)

LOG YOUR BREAKS WITH YOUR SMARTPHONE BY SCANNING THE QR CODES AROUND THE BUILDING

TRACK YOUR PROGRESS ON THE RISE & RECHARGE® APP

SEE HOW WELL YOUR TEAM IS DOING EACH WEEK ON THE LEADERBOARD

03

SEE YOUR RESULTS

AT THE END OF THE STUDY YOU'LL GET A PERSONAL HEALTH REPORT

DON'T WORRY, WE WON'T SHARE ANY PERSONAL HEALTH DATA WITH YOUR EMPLOYER

15

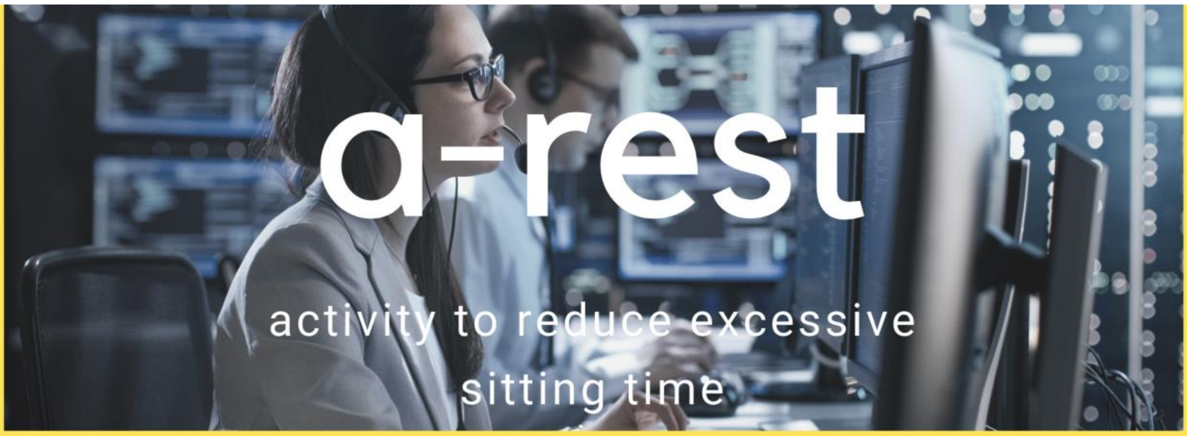

QUESTIONS?

If you have any questions or run into technical issues, contact details for Marsha Brierley and the research team can be found on page 2.

BROUGHT TO YOU BY

University of Bedfordshire

This consultation booklet is adapted from the Active Herts programme (www.activeherts.org.uk)

Active Herts
Your first step to a more active lifestyle

Design by Marsha Brierley 2019

16

Supplementary file 5. Quick response (QR) code and web form example

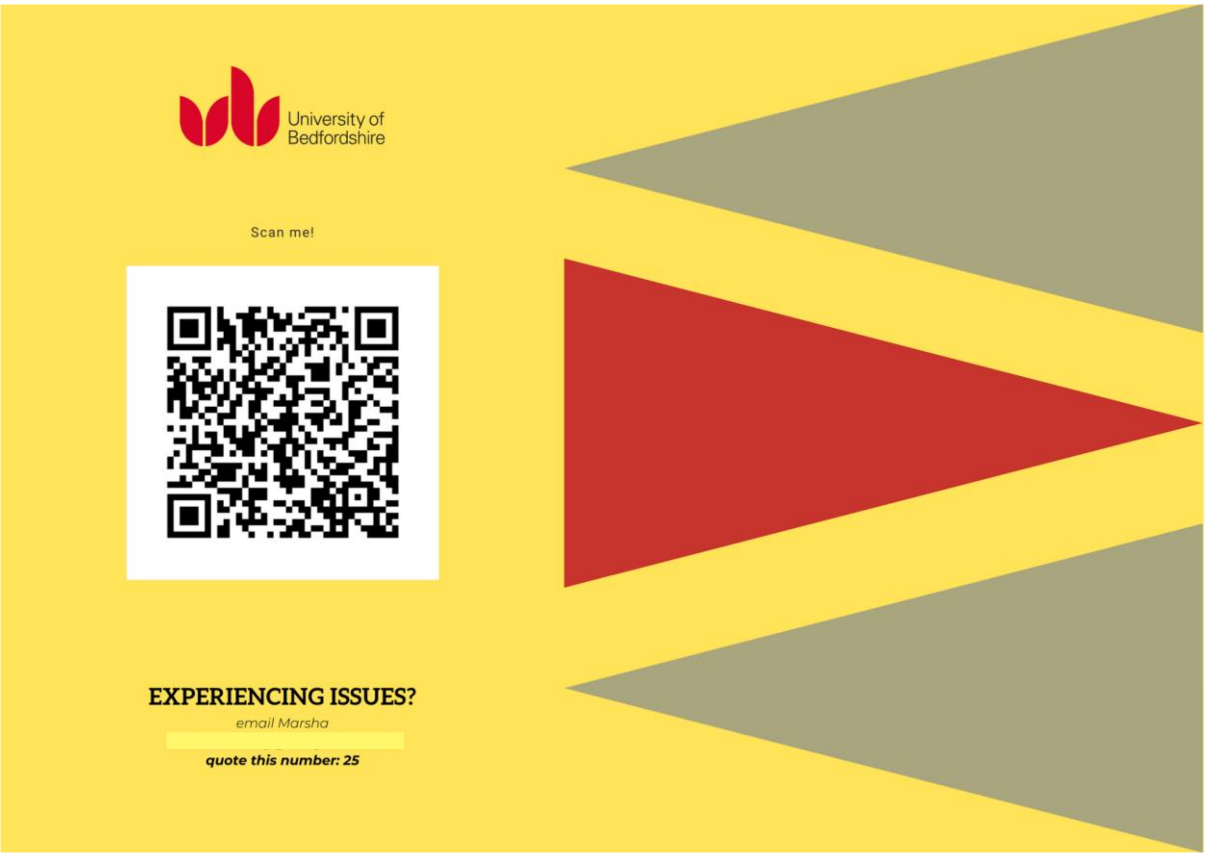

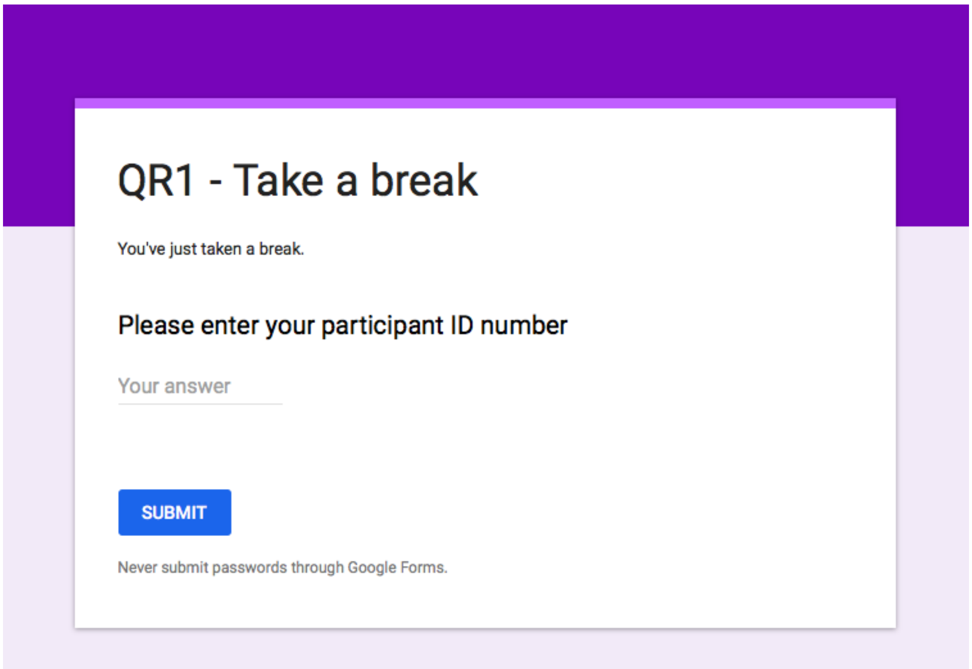

Supplementary file 6. Email template for the A-REST study

Institute for Sport and Physical Activity Research
Polhill Avenue
Bedford
MK41 9EA

A-REST: Activity to Reduce Excessive Sitting

Dear Team,

This is week [X] of the study.

Here is the weekly leaderboard!

Team leaderboard:
15.5pts– Team 1
14pts– Team 2
13pts – Team 3

[Management approval message example – to be personalised]
During my walk through the office this morning I have noticed that many of you have been taking your breaks. Great effort team!

Other personalisation suggestions:
- restating team goals
- ergonomic tips
- sustainability messages (e.g., these changes are not just for the study)
- state approval for participation
- thanks for participating
- invitations to go to lunch/walk/break
- check in with others

Keep up the good work!

[Signature]

Supplementary file 7. Post-intervention interview schedule for the A-REST study mapped to the Capability, Opportunity, Motivation, Behaviour (COM-B) model and Theoretical Domains Framework (TDF) (adapted from Ojo, Bailey, Hewson, & Chater, 2019)

Adapted from Ojo et al. (2019)

**COM-B Component**		**TDF Domain**	**Eliciting Questions**
CAPABILITY	Psychological	Knowledge	*What is your understanding of guidelines from experts about how much sitting time is okay?*
			Prompt – At what point do you think sitting becomes too much?
			Prompt – What do you think are the consequences of sitting for long periods?
			How do you think your sitting time during the intervention compared with this advice?
		Memory, Attention and Decision Processes	*What aspects of the intervention helped you to overcome barriers that might prevent you from breaking up and reducing your sitting at work?*
		Behavioural Regulation	*What else would need to change to help you break up long periods of sitting at work?*
	Physical	Skills	*What things during the intervention still prevented you from breaking up and reducing your sitting time at work?*
OPPORTUNITY	Social	Social influences	*How did the people you work with influence your sitting time?*
			Prompt – How did the people on your team influence you? (e.g., health champions, and including any electronic communications)
			Prompt – How did co-workers not involved in the study influence you?

			How else could your work colleagues and employer help you to break up and reduce your sitting time?
	Physical	Environmental context and Resources	*How did the change in your environment at work influence your sitting behaviour? (e.g., QR codes, computer prompts)*
			How else would your environment need to change to make breaking up and reducing sitting easier for you?
MOTIVATION	Reflective	Beliefs about Capabilities	*How confident were you at the start of the intervention that you could break up your sitting time? On a scale of 1 to 10. Why [insert number] and not 1?*
			How confident are you that you could continue to break up your sitting time going forwards? On a scale of 1 to 10. Why [insert number] and not 1?
		Beliefs about Consequences	*How much benefit do you feel breaking up your sitting time gave you?*
		Goal	*Describe your personal and team goals during the intervention*
		Intention	*How did your determination to break up sitting time progress throughout the intervention?*
		Optimism	*How optimistic were you during the intervention that you could reduce your sitting time? Why is that?*
		Professional/social role and identity	*Thinking about your professional role in policing, what are the positives and negatives of sitting in the workplace?*
			Prompt – How are you perceived by others when you sit, stand, don't sit, don't stand?
			Prompt – How do you perceive yourself when you sit, stand, don't sit, don't stand?
	Automatic	Emotion	*How do you think your mood during the day influenced your sitting patterns?*
		Habits & routines	*What about your habits?*
			How could the intervention address these (mood & habits) to break up and reduce your sitting time at work?

Reinforcement	*In what ways did the intervention reinforce breaking up and reducing sitting time?*

PRACTICABILITY (FIDELITY)

- Did you attend a workshop in the beginning? How useful was it to you?
- Were you aware of the booklet? How useful was it to you?
- Were you aware of the emails?
- Were you aware of the QR code competition?
- Were you aware of where the QR codes were located?
- How useful was the rise & recharge app (to track your behaviour)?
- How did you remember to break up your sitting time (app, kept eye on the clock, etc., alarm or someone else's alarm)?
- How did you find the 8-week timeframe?
- Data collection?
- Were you aware that you would receive a health report at the end?
- Is there anything else you'd like to say about your experience of the intervention, the QR codes, or data collection process?

Supplementary file 8. Table showing changes in daily sitting, standing, and stepping (normalised to a 16-hour waking day) from baseline to end of intervention (n=17).

Variable	Baseline mean	95% CI	End of intervention mean	95% CI	Mean difference	95% CI	P	Effect size (d)
Sitting time (minutes)	630.71	586.57, 674.86	650.76	609.69, 691.83	20.04	-13.64, 53.73	0.23	0.24
Sitting time (%)	65.70	61.10, 70.30	67.79	63.51, 72.07	2.09	-1.42, 5.60	0.23	0.24
Standing time (minutes)	235.72	196.46, 274.97	223.52	186.17, 260.88	-12.19	-36.35, 11.97	0.30	0.16
Standing time (%)	24.55	20.46, 28.64	23.28	19.39, 27.18	-1.27	-3.79, 1.25	0.30	0.16
Time in prolonged sitting bouts (minutes)	384.29	332.30, 436.27	364.91	324.16, 405.67	-19.37	-67.39, 28.64	0.40	0.21
Time in prolonged sitting bouts (%)	40.03	34.61, 45.45	38.01	33.77, 42.26	-2.02	-7.02, 2.98	0.40	0.21
Number of prolonged sitting bouts	6.97	6.17, 7.78	6.81	6.08, 7.54	-0.16	-1.00, 0.67	0.68	0.11
Number of sit-upright transitions	46.08	41.85, 50.31	48.51	44.22, 52.80	2.43	-2.09, 6.95	0.27	0.29
Stepping time (minutes)	93.57	80.30, 106.84	85.72	76.89, 94.54	-7.85	-21.35, 5.65	0.24	0.36
Number of Steps	7863.39	6,641.96, 9,084.81	7027.51	6,170.78, 7,884.24	-835.87	-2,060.15, 388.41	0.17	0.41

NOTE: CI = confidence interval. The analysis was conducted using 17 complete datasets (activPAL data provided both at baseline and follow-up).
